# Measuring *Escherichia* *coli* Gene Expression during Human Urinary Tract Infections

**DOI:** 10.3390/pathogens5010007

**Published:** 2016-01-15

**Authors:** Harry L. T. Mobley

**Affiliations:** Department of Microbiology and Immunology, University of Michigan Medical School, Ann Arbor, MI 48109, USA; hmobley@umich.edu; Tel.: +1-734-764-1466

**Keywords:** *E. coli*, urinary tract infection, *in vivo* gene expression, RNA-seq

## Abstract

Extraintestinal *Escherichia* *coli* (*E. coli*) evolved by acquisition of pathogenicity islands, phage, plasmids, and DNA segments by horizontal gene transfer. Strains are heterogeneous but virulent uropathogenic isolates more often have specific fimbriae, toxins, and iron receptors than commensal strains. One may ask whether it is the virulence factors alone that are required to establish infection. While these virulence factors clearly contribute strongly to pathogenesis, bacteria must survive by metabolizing nutrients available to them. By constructing mutants in all major metabolic pathways and co-challenging mice transurethrally with each mutant and the wild type strain, we identified which major metabolic pathways are required to infect the urinary tract. We must also ask what else is *E. coli* doing *in vivo*? To answer this question, we examined the transcriptome of *E. coli* CFT073 in the murine model of urinary tract infection (UTI) as well as for *E. coli* strains collected and analyzed directly from the urine of patients attending either a urology clinic or a university health clinic for symptoms of UTI. Using microarrays and RNA-seq, we measured *in vivo* gene expression for these uropathogenic *E. coli* strains, identifying genes upregulated during murine and human UTI. Our findings allow us to propose a new definition of bacterial virulence.

## 1. Introduction

In the molecular era of microbial pathogenesis research, virulence genes were first identified by examining transposon mutants or individual gene mutations using *in vitro* assays. Mutants of bacterial pathogens were then assessed in animal models that mimicked human disease. Genome-wide screens were developed whereby genes and proteins that influenced virulence could be identified, including signature-tagged mutagenesis (STM) [1], *in vivo* expression technology (IVET) [2], and *in vivo*-induced antigen technology (IVIAT) [3]. Then individual gene expression was measured by RT-PCR, and, in limited cases, this was done using infected tissue [4]. The advent of microarray technology allowed the estimation of global gene expression under defined culture conditions such as nitrogen limitation [5], oxygenation [6], and osmotic stress [7].

## 2. Importance of Measuring Bacterial Gene Expression in the Host

In the last decade, a limited number of investigators employed microarrays to assess genome-wide gene expression of pathogens in animal models of infection. These included study of *Borrelia burgdorferi*, *Burkholderia pseudomallei*, *Campylobacter jejuni*, *Escherichia coli*, *Helicobacter pylori*, *Listeria monocytogenes*, *Mycobacterium* spp., *Mycoplasma hyopneumoniae*, *Streptococcus pyogenes*, and *Vibrio cholerae*, in mice and rats [8,9,10,11,12,13,14,15,16], gerbils [17], rabbit ileal loops [18,19], hamsters [20], and pigs [21]. Among these studies, our group measured *in vivo* gene expression of uropathogenic *E. coli* (UPEC) collected directly from urine of experimentally infected mice using the murine model of ascending urinary tract infection (UTI) [12]. We extended studies to humans by determining the transcriptomes of *E. coli* directly from the urine of patients with “complicated” UTI [22] and in otherwise healthy women with “uncomplicated” UTI using the technique of RNA-Seq [23]. Overall, only a few research groups worldwide have measured genome-wide gene expression during a human infection with *V. cholera* [24], *Pseudomonas aeruginosa* [25], *M. tuberculosis* [26], and *E. coli* [22,23].

Taken together, these techniques provided a broad view of virulence. Close examination of the effect on virulence of mutation of individual bacterial genes and operons in animal models, along with complementation studies defined the traditional assessment of virulence. However, other factors are required for colonization and infection. Critically, metabolism of pathogens must match available nutrients and proper levels of oxygenation or lack thereof to survive and thrive in the host. Thus, the contribution of bacterial metabolism must be added to the study of virulence to craft a complete picture.

## 3. Lessons from *E. coli* Help Redefine Bacterial Virulence

To redefine the meaning of virulence of bacterial pathogens, let’s look carefully at one versatile pathogen. Over millions of years, *E. coli* pathotypes have developed by horizontal gene transfer of foreign DNA into commensal strains by conjugation, transduction, and transformation. For example, enterohemorrhagic *E. coli* received the Shiga toxin gene via transduction and the LEE (locus of enterocyte effacement) pathogenicity island probably by conjugation to form this pathotype [27]. Other intestinal and extraintestinal pathotypes have also developed. For example, we can have diarrhea at least six different ways caused by enterotoxigenic, enteropathogenic, enterohemmorhagic, enteroaggregative, enteroinvasive and diffuse-adherent *E. coli*, each with its own mechanism of pathogenesis. Beyond the intestinal tract, extraintestinal *E. coli* causes urinary tract infection and meningitis in humans, and lung infection in birds [27]. So let’s look at one of these pathotypes, uropathogenic *E. coli,* to explore our new definition of virulence.

## 4. The ExPEC Pathotype Helps Make the Case

**Figure 1:**
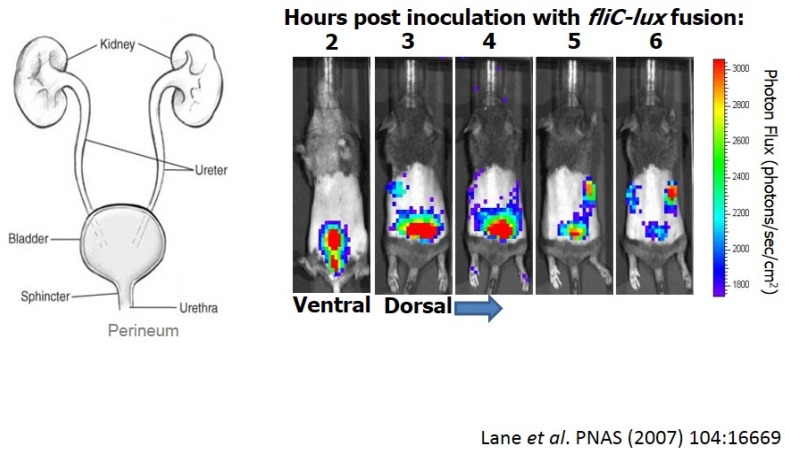
Imaging of uropathogenic *E. coli* during urinary tract infection (UTI).

*E. coli* causes ~80% of ascending urinary tract infections in otherwise healthy women [28]. First, we have fecal contamination of the periurethral area, and then bacteria ascend into the bladder causing cystitis (Figure 1-left panel). These infections of the lower urinary tract, in some cases, may ascend to the kidneys eliciting acute pyelonephritits, possibly leading to bacteremia and sepsis. Thus, as *E. coli* establishes infection, bacteria encounter numerous environments that would likely require significantly different patterns of gene expression to survive.

We can visualize this infection in mice colonized with *E. coli* CFT073 expressing a flagellin-*lux* fusion (Figure 1-right panel). After transuretheral inoculation of mice (ventral view), we can follow the infection for six hours using whole animal imaging [29]. When the flagellin gene (*fliC*) is transcribed, we see a burst of light. Looking at a dorsal view, we can see that these bacteria ascend the ureters to the kidneys just within a couple of hours. In some cases, bacteria can cross epithelial and endothelial barriers into the bloodstream.

These bacteria have a potent virulence arsenal including up to twelve different fimbrial adhesins [30]. The most notable is P fimbriae, which binds the P blood group antigen, a glycosphingolipid expressed on the surface of kidney epithelial cells. Six O serotypes (antigenic variants of LPS) cause three fourths of infections, so these strains may be relatively clonal (however, serotypes are only one trait that define strains). They synthesize capsules (K antigen) that evoke serum resistance. They’re chemotactic and motile by flagella. They make many iron and heme receptors, and exotoxins such as hemolysin, cytotoxic necrotizing factor, cytolethal distending toxin, and several autotransported proteases such as Sat.

Another way to define UPEC strains is to examine acquisition of genomic or pathogenicity islands. In one example, if we hybridize genomic DNA from representative fecal/commensal strains, cystitis strains, and pyelonephritis strains to a microarray of *E. coli* CFT073 (the most cited prototype UPEC strain) from gene 1 through gene 5364, we see that *E. coli* CFT073 contains significant stretches of DNA not present in other strains, particularly fecal strains. Indeed, CFT073, carries 13 pathogenicity islands, each of 30–100 kb, that have been inserted around chromosome, comprising about 12% of the genome [31,32]. They’ve added accessory genes to the “base model” *E. coli* that may increase fitness of a strain in the urinary tract.

**Figure 2:**
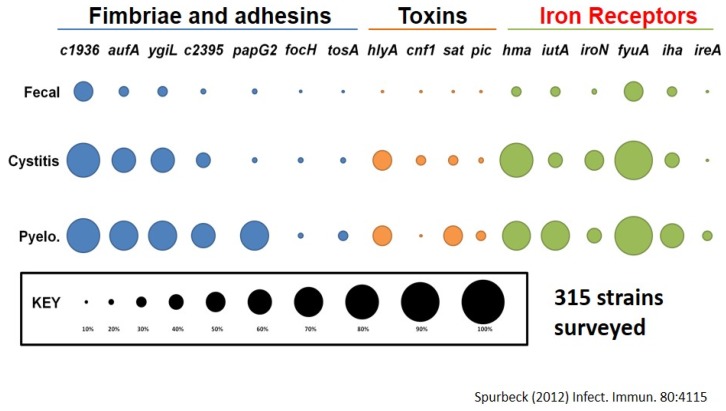
Prevalence of virulence genes found on pathogenicity islands.

If we look at the prevalence of those genes found in a survey of 315 strains, from fecal strains, cystitis strains, and pyelonephritis strains, we note that as *E. coli* goes higher in the urinary tract, it is considered more virulent and is more likely to have these genes. For example, about 70% of pyelonephritis strains (ones infecting kidneys) have a particular fimbria called “Auf” (another UPEC fimbria) found in <20% of fecal strains. We see this relationship also with other fimbriae, toxins and iron acquisition systems (Figure 2) [33,34].

## 5. What Else Should We Consider to Define Bacterial Virulence?

At this stage we must ask whether these virulence genes are all that is required for infection. The answer is, of course not. There are other factors including metabolic pathways that drive fermentation and oxidative phosphorylation. We have glycolysis, gluconeogenesis, the TCA cycle, pentose phosphate pathway, and the Entner-Duodoroff pathway. We must know what these bacteria eat during colonization of the urinary tract. What is available to them? *A priori*, you would assume that *E. coli* needs all of these heralded cycles. To test this idea, we made single mutations that specifically knocked out each one of these cycles and then inoculated these mutants into the bladders of mice and quantified bacteria 48 h later. Mutants with defects in the TCA cycle or gluconeogenesis had impaired fitness during UTI [35,36]. That’s not so surprising. But, glycolysis, pentose phosphate, and Entner-Duodoroff pathways are all dispensable. Constructs in which these pathways are knocked out, colonize the urinary tract as well as the wild type strain. Peptide transporters like DppA and OppA are induced in urine, and required for fitness during infection [36]. So we surmised that amino acids and peptides are the primary carbon source for *E. coli* during infection of the urinary tract. Peptides are taken up, converted into amino acids and then oxaloacetate enters the TCA cycle to boost oxidative phosphorylation and gluconeogenesis to make glucose. Bacteria don’t require exogenous glucose. Indeed, glucose is not usually present in the urinary tract in high amounts unless under diabetic conditions. So this was a very surprising finding—that you need just a few of the major metabolic pathways during infection.

## 6. Measuring Global Gene Expression during Bacterial Infection

**Figure 3:**
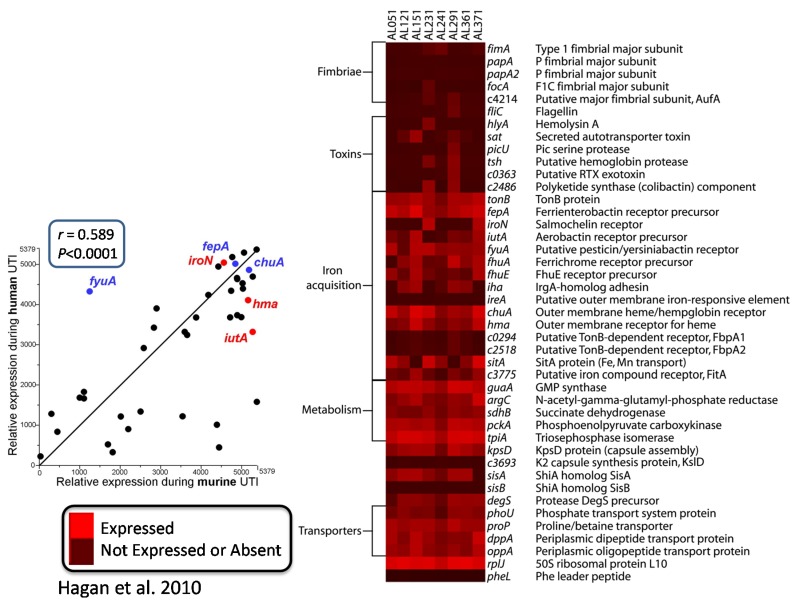
Virulence gene expression by *E. coli* in urine of patients with UTI.

That prompted the question: what else is *E. coli* doing *in vivo*? To answer this, we looked at the *in vivo* transcriptome several ways. We collected bacteria from the urine of infected CBA mice, immediately stabilized the RNA [12], or from women attending the Urology Clinic with urinary tract infection (these tended to be more “complicated” infections defined as occurring in individuals with structural abnormalities of the urinary tract or those with urinary catheters in place or in immunocompromised patients) [22]. Bacterial RNA was isolated and converted into cDNA, and then we hybridized it to the *E. coli* CFT073 microarray (Figure 3). For eight strains from patients, listed across the top of the heat map, “red” is expressed and “black” is not expressed or absent, and virulence factor genes measured were for fimbriae, toxins, iron acquisition, capsule, metabolism genes, and transporters. If we compare relative expression during human UTI *versus* relative expression during murine UTI, we have a very good correlation (r = 0.589; *p* < 0.0001), especially with the iron acquisition proteins, which are highly expressed in both mice and humans. In humans, however, fimbrial genes are not as well expressed by bacteria collected in the urine (perhaps because adhering bacteria are not released into the urine).

These studies were very informative, but the comparison was not ideal, because we were comparing expression of the eight strains from the urology clinic using the genome of another strain, *E. coli* CFT073. Genes may differ in relatedness between the clinical strains and strain CFT073 resulting in poor hybridization, leading to confusion as to whether a gene is absent, or just not expressed. We wanted to overcome this uncertainty. We then asked what the *E. coli* was doing during UTI in women with uncomplicated infections (these occur in otherwise healthy individuals) by using RNA-seq in naturally occurring human UTIs [23].

For this study, 86 women attending the University Health Service with symptoms of cystitis were given informed consent and enrolled in the study [23]. Urine was collected, sampled for culture, and stabilized immediately within 10 min in *RNAprotect* (in this case, up to 200 mL of *RNAprotect/*sample) acknowledging that we didn’t know whether bacteria were in the samples in significant numbers or at all. Forty-two women, about half, had bacteria at ≥10^5^ colony-forming units/mL, and 38 were positive for *E. coli*. We also isolated those strains and subjected them to genomic sequencing. RNA was isolated and, for the five strains with the most abundant RNA preparations, was subjected to Illumina sequencing to determine the transcriptome for five strains. We also cultured each isolate in LB or in urine from age-matched volunteers and conducted RNAseq. By phylogenetic analysis, these strains were UPEC strains in the B2 and D phylogenetic groups, lining up well with prototype UPEC strains CFT073, 536, UTI89 (all B2) and UMN26 (D). Also consistent with UPEC strains, the genome sizes were 8%–15% larger than *E. coli* K12, suggesting that they have quite a few of the pathogenicity islands inserted into the genomes.

**Figure 4:**
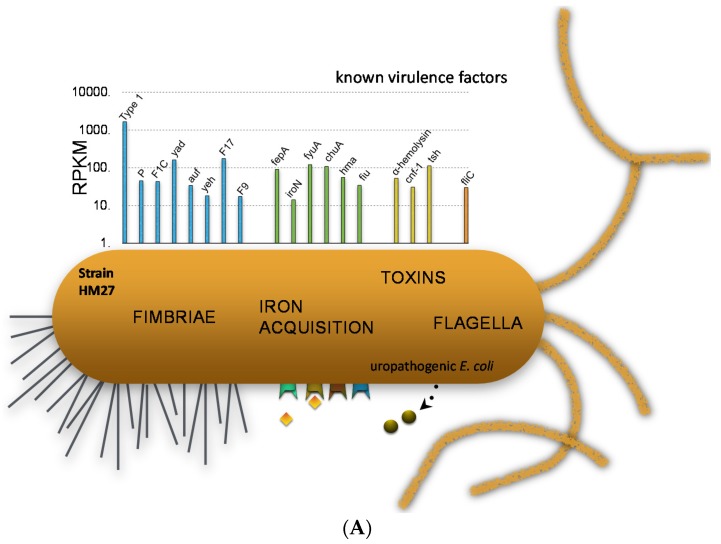

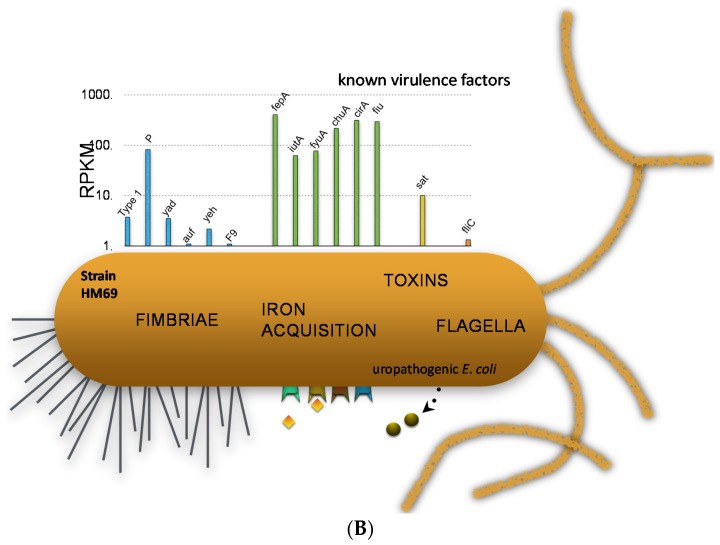
*E. coli* virulence gene transcript levels during human UTI. (**A**) Results from *E. coli* HM27; (**B**) Results from *E. coli* HM69.

We can examine the expression of traditional virulence factor genes in two strains to demonstrate specific points (Figure 4). In one strain, we can see that type 1 fimbriae were very highly expressed. P fimbriae and F1C fimbriae were also expressed, but more weakly. Iron acquisition proteins are highly expressed because urine is iron-limiting, and so these Fur-regulated iron genes are very highly expressed [23]. Toxin genes such as hemolysin, CNF, and proteases and also flagella are expressed somewhat. However, if we look at another strain, we see a different situation where the type 1 fimbriae are very poorly expressed but P fimbriae are expressed well and iron acquisition genes are even higher than for the first strain.

Thus, there is heterogeneity between strains in virulence gene expression *in vivo*. Importantly, expression of type 1 fimbria is critical to consider because it’s expression is essential in the murine model, but for the most part, *fim* genes are variably expressed in human UTI (sometimes ON and sometimes OFF). The promoter for type 1 fimbria resides on an invertible element, and can invert using recombinases and then is turned ON and OFF. So it’s phase variable. Using an invertible element assay, we determined for 21 strains in the urine of women with UTI, six strains were ON (the whole population expressed type 1 fimbriae); for 12 strains, the whole population was OFF; and three strains were mixed (population included both ON and OFF) [23].

## 7. Comparative Transcriptomics Reveal Host-Induced Bacterial Genes

Using comparative transcriptomics, we examined the expression or abundance of RNA in urine of UTI patients *versus* the same bacterium cultured *in vitro* in urine from age-matched, healthy volunteers [23]. If we look at the ratio of these transcripts in UTI over urine, we can identify transcripts that are highly expressed in the host but not in urine. What are the host-specific genes? Averaging data for five strains, host-induced genes were those encoding import systems for sulphonate, nickel, phosphonate, taurine, and potassium, and copper efflux, osmoprotection, and colanic acid synthesis. These are representative of UTI-specific genes. That is, something about the host (not just urine) is turning these genes on. Of course, bacteria were also synthesizing fimbriae, toxins, iron acquisition systems and other virulence factors, but they’re also transcribing these genes during *in vitro* culture in human urine. If we compare samples from UTI *versus* urine, we see that these genes were highly expressed under those conditions. Of course, one must verify this by qPCR, and they are on average 32-fold more highly expressed during infection than culture. For each one of those genes, we needed to verify their involvement in virulence. We made clean mutations, mixed mutant with wild type CFT073, transurethrally inoculated 10^8^ into the mouse, and 48 h later, quantitatively cultured the bladder and the kidneys to calculate the competitive index (mutant CFU/wild type CFU). For copper export, we saw that they were indeed outcompeted and less fit when you knock out the *cus* genes. For ethanolamine uptake, bacteria appear to be metabolizing ethanolamine in the epithelial cells. Formate hydrogen lyase, which is induced under anaerobiosis, indicates that there’s an anaerobic environment sometime during infection, and finally, bacteria are importing nickel, sulfonate, ethanolamine, potassium, phosphonate and taurine at high rates and effluxing copper ions.

## 8. Different Bacterial Genes are Required for Different Host Settings

UTIs may, in some cases, progress from cystitis to pyelonephritis and then *E. coli* can break through the tubules and capillaries in the kidney into the bloodstream. To identify genes important for development of bacteremia, we can conduct transposon-directed insertion site sequencing, undertaking a simultaneous screen of 360,000 transposon mutants by inoculating the mutant pool into the tail vein of a mouse [37]. We recover those bacteria from the spleen and then sequence the genomic DNA from the pool to see which mutants are underrepresented in the bloodstream (that is, genes that provide a fitness advantage during bloodstream infection). For example, in the inoculum (input), a *sensitivity to antimicrobial peptide* (*sap*) operon gene had 622 insertions, but what we got back from the spleen (output) only contained 91 insertions (fitness factor of 6.8). We investigated genes with fitness factors 2 standard deviations above the mean. The top 11 mutants selected for validation were in poly N-acetyl glucosamine production, another iron receptor, a zinc peptidase, a type IV pilus, oligopeptide uptake, resistance to antimicrobial peptides, and two serine proteases. These mutants were significantly outcompeted by wild type during bacteremia.

## 9. Redefining Bacterial Virulence

Our conventional view of virulence focused on adhesins, iron acquisition, toxins, secretion, and motility; bacteria relied on genes not found in commensal strains such as on horizontally transferred pathogenicity-associated islands. Attenuation of mutants of these genes in animal models, followed by restoration of virulence by complementation defined traditional virulence [38]. But now we must consider what metabolic pathways are in play. What’s for dinner? How are the bacteria metabolizing these molecules to colonize a particular body site? Which import and export systems are active during infection? A mutation of these systems should reduce that fitness. We must measure transcription under relevant conditions, and, for human pathogens, that’s human infection when possible rather than artificial systems. Human infection-specific gene expression in this case was measured by RNA-seq. Finally, we can hypothesize that virulence is the sum of required metabolic pathways, the traditional virulence determinants, and upregulated transport systems and other indispensable functions.

## References

[1] Hensel M., Shea J.E., Gleeson C., Jones M.D., Dalton E., Holden D.W. (1995). Simultaneous identification of bacterial virulence genes by negative selection. Science.

[2] Slauch J.M., Mahan M.J., Mekalanos J.J. (1994). *In vivo* expression technology for selection of bacterial genes specifically induced in host tissues. Methods Enzymol..

[3] Handfield M., Brady L.J., Progulske-Fox A., Hillman J.D. (2000). Iviat: A novel method to identify microbial genes expressed specifically during human infections. Trends Microbiol..

[4] Shimoyama T., Everett S.M., Dixon M.F., Axon A.T., Crabtree J.E. (1998). Chemokine mrna expression in gastric mucosa is associated with helicobacter pylori caga positivity and severity of gastritis. J. Clin. Pathol..

[5] Hua Q., Yang C., Oshima T., Mori H., Shimizu K. (2004). Analysis of gene expression in *Escherichia coli* in response to changes of growth-limiting nutrient in chemostat cultures. Appl. Environ. Microbiol..

[6] Salmon K., Hung S.P., Mekjian K., Baldi P., Hatfield G.W., Gunsalus R.P. (2003). Global gene expression profiling in *Escherichia coli* k12. The effects of oxygen availability and FNR. J. Biol. Chem..

[7] Cheung K.J., Badarinarayana V., Selinger D.W., Janse D., Church G.M. (2003). A microarray-based antibiotic screen identifies a regulatory role for supercoiling in the osmotic stress response of *Escherichia coli*. Genome Res..

[8] Camejo A., Buchrieser C., Couve E., Carvalho F., Reis O., Ferreira P., Sousa S., Cossart P., Cabanes D. (2009). *In vivo* transcriptional profiling of listeria monocytogenes and mutagenesis identify new virulence factors involved in infection. PLoS Pathog..

[9] Graham M.R., Virtaneva K., Porcella S.F., Gardner D.J., Long R.D., Welty D.M., Barry W.T., Johnson C.A., Parkins L.D., Wright F.A. (2006). Analysis of the transcriptome of group a streptococcus in mouse soft tissue infection. Am. J. Pathol..

[10] Haugen B.J., Pellett S., Redford P., Hamilton H.L., Roesch P.L., Welch R.A. (2007). *In vivo* gene expression analysis identifies genes required for enhanced colonization of the mouse urinary tract by uropathogenic *Escherichia coli* strain cft073 dsda. Infect. Immun..

[11] Revel A.T., Talaat A.M., Norgard M.V. (2002). DNA microarray analysis of differential gene expression in borrelia burgdorferi, the lyme disease spirochete. Proc. Natl. Acad. Sci. USA.

[12] Snyder J.A., Haugen B.J., Buckles E.L., Lockatell C.V., Johnson D.E., Donnenberg M.S., Welch R.A., Mobley H.L. (2004). Transcriptome of uropathogenic *Escherichia coli* during urinary tract infection. Infect. Immun..

[13] Stintzi A., Marlow D., Palyada K., Naikare H., Panciera R., Whitworth L., Clarke C. (2005). Use of genome-wide expression profiling and mutagenesis to study the intestinal lifestyle of campylobacter jejuni. Infect. Immun..

[14] Talaat A.M., Lyons R., Howard S.T., Johnston S.A. (2004). The temporal expression profile of mycobacterium tuberculosis infection in mice. Proc. Natl. Acad. Sci. USA.

[15] Talaat A.M., Ward S.K., Wu C.W., Rondon E., Tavano C., Bannantine J.P., Lyons R., Johnston S.A. (2007). Mycobacterial bacilli are metabolically active during chronic tuberculosis in murine lungs: Insights from genome-wide transcriptional profiling. J. Bacteriol..

[16] Williams D.L., Torrero M., Wheeler P.R., Truman R.W., Yoder M., Morrison N., Bishai W.R., Gillis T.P. (2004). Biological implications of mycobacterium leprae gene expression during infection. J. Mol. Microbiol. Biotechnol..

[17] Scott D.R., Marcus E.A., Wen Y., Oh J., Sachs G. (2007). Gene expression *in vivo* shows that helicobacter pylori colonizes an acidic niche on the gastric surface. Proc. Natl. Acad. Sci. USA.

[18] Nielsen A.T., Dolganov N.A., Otto G., Miller M.C., Wu C.Y., Schoolnik G.K. (2006). Rpos controls the vibrio cholerae mucosal escape response. PLoS Pathog..

[19] Xu Q., Dziejman M., Mekalanos J.J. (2003). Determination of the transcriptome of vibrio cholerae during intraintestinal growth and midexponential phase *in vitro*. Proc. Natl. Acad. Sci. USA.

[20] Tuanyok A., Tom M., Dunbar J., Woods D.E. (2006). Genome-wide expression analysis of burkholderia pseudomallei infection in a hamster model of acute melioidosis. Infect. Immun..

[21] Madsen M.L., Puttamreddy S., Thacker E.L., Carruthers M.D., Minion F.C. (2008). Transcriptome changes in mycoplasma hyopneumoniae during infection. Infect. Immun..

[22] Hagan E.C., Lloyd A.L., Rasko D.A., Faerber G.J., Mobley H.L. (2010). *Escherichia coli* global gene expression in urine from women with urinary tract infection. PLoS Pathog..

[23] Subashchandrabose S., Hazen T.H., Brumbaugh A.R., Himpsl S.D., Smith S.N., Ernst R.D., Rasko D.A., Mobley H.L. (2014). Host-specific induction of *Escherichia coli* fitness genes during human urinary tract infection. Proc. Natl. Acad. Sci. USA.

[24] Larocque R.C., Harris J.B., Dziejman M., Li X., Khan A.I., Faruque A.S., Faruque S.M., Nair G.B., Ryan E.T., Qadri F. (2005). Transcriptional profiling of vibrio cholerae recovered directly from patient specimens during early and late stages of human infection. Infect. Immun..

[25] Son M.S., Matthews W.J., Kang Y., Nguyen D.T., Hoang T.T. (2007). *In vivo* evidence of pseudomonas aeruginosa nutrient acquisition and pathogenesis in the lungs of cystic fibrosis patients. Infect. Immun..

[26] Timm J., Post F.A., Bekker L.G., Walther G.B., Wainwright H.C., Manganelli R., Chan W.T., Tsenova L., Gold B., Smith I. (2003). Differential expression of iron-, carbon-, and oxygen-responsive mycobacterial genes in the lungs of chronically infected mice and tuberculosis patients. Proc. Natl. Acad. Sci. USA.

[27] Kaper J.B., Nataro J.P., Mobley H.L. (2004). Pathogenic *Escherichia coli*. Nat. Rev. Microbiol..

[28] Foxman B., Barlow R., D’Arcy H., Gillespie B., Sobel J.D. (2000). Urinary tract infection: Self-reported incidence and associated costs. Ann. Epidemiol..

[29] Lane M.C., Alteri C.J., Smith S.N., Mobley H.L. (2007). Expression of flagella is coincident with uropathogenic *Escherichia coli* ascension to the upper urinary tract. Proc. Natl. Acad. Sci. USA.

[30] Welch R.A., Burland V., Plunkett G., Redford P., Roesch P., Rasko D., Buckles E.L., Liou S.R., Boutin A., Hackett J. (2002). Extensive mosaic structure revealed by the complete genome sequence of uropathogenic *Escherichia coli*. Proc. Natl. Acad. Sci. USA.

[31] Lloyd A.L., Henderson T.A., Vigil P.D., Mobley H.L. (2009). Genomic islands of uropathogenic *Escherichia coli* contribute to virulence. J. Bacteriol..

[32] Lloyd A.L., Rasko D.A., Mobley H.L. (2007). Defining genomic islands and uropathogen-specific genes in uropathogenic *Escherichia coli*. J. Bacteriol..

[33] Spurbeck R.R., Dinh P.C., Walk S.T., Stapleton A.E., Hooton T.M., Nolan L.K., Kim K.S., Johnson J.R., Mobley H.L. (2012). *Escherichia coli* isolates that carry vat, fyuA, chuA, and yfcV efficiently colonize the urinary tract. Infect. Immun..

[34] Spurbeck R.R., Stapleton A.E., Johnson J.R., Walk S.T., Hooton T.M., Mobley H.L. (2011). Fimbrial profiles predict virulence of uropathogenic *Escherichia coli* strains: Contribution of ygi and yad fimbriae. Infect. Immun..

[35] Alteri C.J., Himpsl S.D., Mobley H.L. (2015). Preferential use of central metabolism *in vivo* reveals a nutritional basis for polymicrobial infection. PLoS Pathog..

[36] Alteri C.J., Smith S.N., Mobley H.L. (2009). Fitness of *Escherichia coli* during urinary tract infection requires gluconeogenesis and the tca cycle. PLoS Pathog..

[37] Subashchandrabose S., Smith S.N., Spurbeck R.R., Kole M.M., Mobley H.L. (2013). Genome-wide detection of fitness genes in uropathogenic *Escherichia coli* during systemic infection. PLoS Pathog..

[38] Falkow S. (1988). Molecular koch’s postulates applied to microbial pathogenicity. Rev. Infect. Dis..

